# Acute Clinical Decline in Anterior Convexity Dural Arteriovenous Fistula Highlighting the Role of Bridging Vein Anatomy: A Case Report and Literature Review

**DOI:** 10.7759/cureus.98227

**Published:** 2025-12-01

**Authors:** Misaki Kamogawa, Ryosuke Suzuki, Tomoyuki Yokose, Yasunobu Nakai, Tetsuya Yamamoto

**Affiliations:** 1 Department of Neurosurgery, Odawara Municipal Hospital, Odawara, JPN; 2 Department of Neurosurgery, Yokohama City University Graduate School of Medicine, Yokohama, JPN; 3 Department of Pathology, Odawara Municipal Hospital, Odawara, JPN

**Keywords:** borden type ⅲ, convexity dural arteriovenous fistula, intracerebral hemorrhage, lesion location, pial venous reflux

## Abstract

Convexity dural arteriovenous fistulas (dAVF) are a rare arteriovenous shunt disorder, typically classified as Borden type Ⅲ with cortical venous reflux. We describe the possibility that variations in venous drainage patterns related to lesion location may influence the clinical course of convexity dAVF. An 80-year-old man presented with progressive motor aphasia and dysarthria. Magnetic resonance fluid-attenuated inversion recovery imaging demonstrated hyperintensity in the left frontal lobe. Cerebral angiography revealed a Borden type Ⅲ convexity dAVF at the coronal suture level with retrograde cortical venous drainage. The following day, he experienced a seizure, accompanied by intracerebral hemorrhage (ICH) and worsening venous congestion. Given the rapid progression, middle meningeal artery embolization was performed, followed by surgical shunt disconnection. Postoperatively, motor aphasia and higher cortical dysfunction improved markedly. Histopathological examination revealed no occlusion or remnant of dural veins connected to a venous lake or superior sagittal sinus (SSS), indicating a shunt formation within a normal dural arteriovenous network. The shunt was located near the coronal suture, where bridging veins to the SSS are anatomically sparse. Without compensatory drainage, pial venous reflux (PVR) predominated, resulting in ICH due to worsening venous congestion. These findings suggest that in convexity dAVF, anatomical differences in bridging vein distribution may lead to distinct drainage patterns between anterior and parietal lesions, even within the same Borden type Ⅲ. In particular, cases with predominant PVR may experience rapid clinical deterioration, necessitating prompt intervention.

## Introduction

Dural arteriovenous fistulas (dAVFs) are vascular malformations characterized by abnormal shunts between meningeal arteries and dural venous sinuses, meningeal veins, or cortical veins [1]. Convexity dAVFs are relatively uncommon and often exhibit direct retrograde drainage into cortical veins [2,3]. Cortical venous reflux (CVR), defined as retrograde drainage into cortical veins, is a major determinant of the aggressive clinical behavior, including intracranial hemorrhage, non-hemorrhagic neurological symptoms, and even death [4]. Accordingly, timely and appropriate therapeutic intervention is essential for improving clinical outcomes [3].

Convexity dAVFs arise from the dural surface over the cerebral convexity, remote from the superior sagittal sinus (SSS), and typically drain into superficial cortical veins. Although the clinical features of convexity dAVFs have been well described, potential differences in pathophysiology depending on lesion location or patterns of CVR have not been fully discussed. Hemorrhagic presentations of convexity dAVFs include intracerebral hemorrhage (ICH), subdural hematoma (SDH), and subarachnoid hemorrhage. In the literature, most reported convexity dAVFs are located in the parietal region (parietal convexity dAVFs). Several of these presented with SDH, likely due to rupture of bridging or dural veins draining into the SSS [5-7]. In contrast, anterior convexity lesions are rarely reported, and their clinical characteristics and natural history remain poorly understood.

We present a case of an anterior convexity dAVF that led to ICH with seizures and a rapid clinical decline. Convexity dAVFs, in which fistulous flow refluxes directly into cortical veins, leading to venous hypertension and a high risk of hemorrhage, are generally classified as Borden type Ⅲ [1]. However, their clinical presentation may vary depending on the shunt location. In particular, anterior lesions located anteriorly may become symptomatic and progress aggressively. This case highlights the anatomical characteristics of anterior convexity dAVFs and emphasizes the importance of early therapeutic intervention. This case report was previously presented as a poster at the 84th Annual Meeting of the Japan Neurosurgical Society on October 30, 2025.

## Case presentation

**Figure 1:**
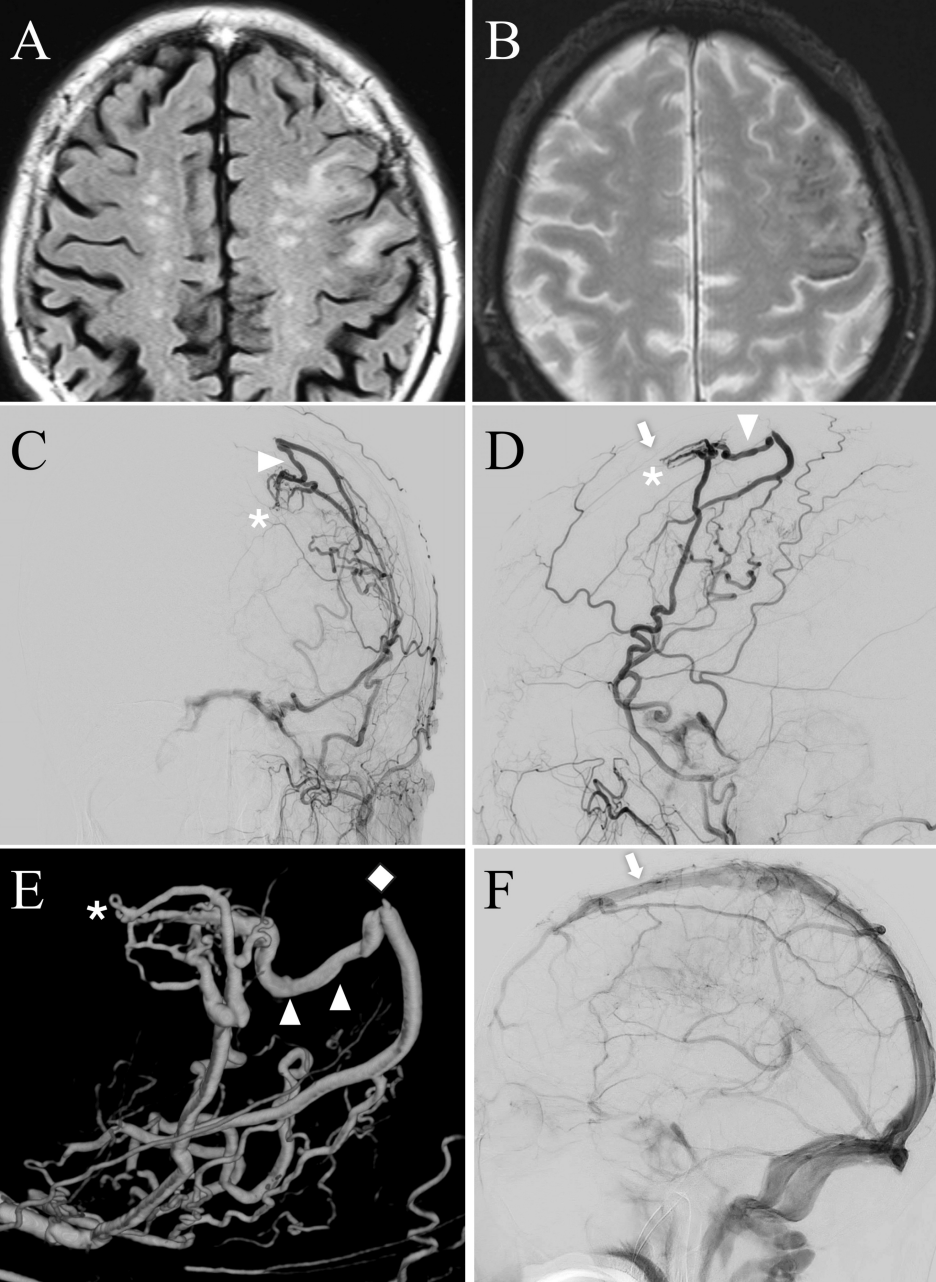
Admission magnetic resonance imaging (A) and (B) and cerebral angiography (C)–(F). The fluid-attenuated inversion recovery image demonstrates focal edema in the left middle frontal and precentral gyri (A). The T2*-weighted image reveals multiple microbleeds in the left middle frontal gyrus that are inconspicuous on computed tomography (CT) (B). Left external carotid artery angiography—anteroposterior (C) and lateral (D) views—shows a convexity dural arteriovenous fistula (dAVF) supplied by the anterior convexity branch of the left middle meningeal artery (MMA), with cortical venous reflux (white arrowheads) draining retrogradely into the superficial middle cerebral veins. The shunt point (asterisk) is located near the coronal suture (white arrow). Three-dimensional rotational angiography viewed from the superolateral aspect clearly depicts the vascular architecture (E), demonstrating occlusion of a bridging vein posterior to the shunt, likely draining toward the superior sagittal sinus (SSS) (white diamond). Left internal carotid angiography shows no developed bridging vein near the coronal suture (white arrow) and demonstrates congestion of the medullary veins (F).

An 80-year-old man with no history of head trauma or prior neurosurgery was brought to the emergency department because of a suspected mild disturbance of consciousness. No focal neurological deficits or abnormal radiographical findings were identified, and he was discharged after a short period of observation. However, three weeks later, he presented to our department with progressively worsening speech impairment and dysarthria. On admission, his Glasgow Coma Scale score was E4V4M6, with mild motor aphasia but no motor or sensory deficits [8]. Brain magnetic resonance imaging fluid-attenuated inversion recovery sequences revealed hyperintensity in the middle frontal and precentral gyri (Figure 1A). T2*-weighted imaging revealed multiple microbleeds in the same region, which were inconspicuous on computed tomography (CT) (Figure 1B). Magnetic resonance angiography revealed a prominently enlarged left middle meningeal artery (MMA). Cerebral angiography demonstrated a convexity dAVF with a shunt point located in the dura mater over the left frontal convexity, supplied predominantly by the left MMA but also receiving feeders from the right MMA. The shunt drained retrogradely through a bridging vein directly into cortical veins and subsequently into the superficial middle cerebral vein, consistent with Borden type Ⅲ and Cognard type Ⅲ classifications (Figures 1C-1F). No alternative venous drainage route toward the SSS was identified in the anterior frontal region, indicating that the refluxed flow appeared to follow a single cortical venous pathway.

**Figure 2:**
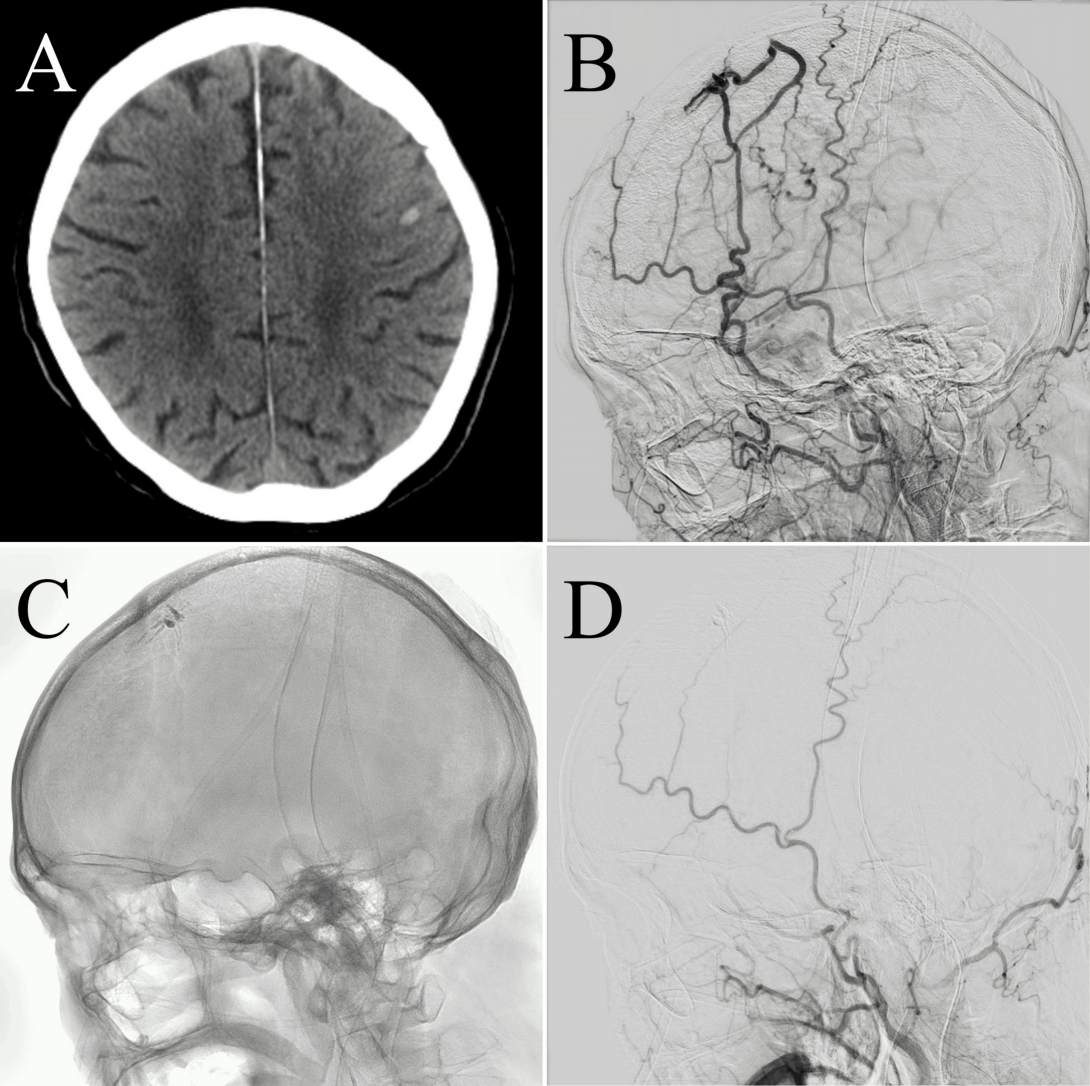
Post-admission imaging (A) and (B) and treatment course (C) and (D). Computed tomography (CT) obtained the day after admission shows an intracerebral hemorrhage (ICH) in the left frontal lobe (A). Repeat left external carotid angiography (lateral view) reveals a pseudophlebitic pattern with marked worsening of venous congestion compared with the previous day's findings (B). Intraoperative angiography following n-butyl cyanoacrylate (NBCA) injection into the left middle meningeal artery (MMA) is shown (C). Postoperative left external carotid angiography demonstrates near-complete resolution of the dural arteriovenous fistula (dAVF) (D).

**Figure 3:**
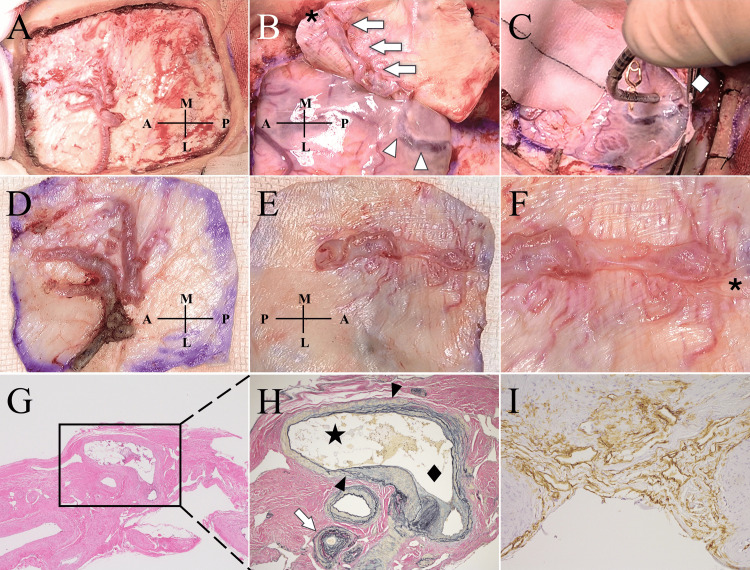
Intraoperative (A)–(C) and histopathological (D)–(I) findings. The dural surface beneath the frontoparietal craniotomy shows a hypertrophied middle meningeal artery (MMA) as the main feeder (A). Shunt point (asterisk) is identified on the inner dural surface, with the draining dural vein (white arrows) connecting to the cortical vein (white arrowheads) (B). The cortical vein is occluded at its dural entry point posterior to the shunt (white diamond), and the bridging vein is presumed to drain toward the superior sagittal sinus (SSS) (C). Outer surface of the dura mater (D) and inner surface of the dura mater (E) are shown. An enlarged view around the shunt point (F) shows fine branches of the MMA converging on the shunt (asterisk), and the draining intradural vein is markedly dilated. The arterial wall shows an intimal thickening with myxoid degeneration (G), and no perivascular inflammatory infiltrates are observed (hematoxylin and eosin stain, ×4). An arteriovenous connection (black arrowheads) between a dural artery (star) and a dural vein (black diamond) is observed (H). Multilaminar elastic fibers (white arrows) with eccentric intimal thickening are seen in the veins surrounding the shunt. No evidence of dural veins connected to the SSS or a venous lake is identified in the most medial section (Alcian blue and Elastica van Gieson stain, ×10). Immunohistochemistry for CD34 highlights proliferating capillary channels within the dura adjacent to the shunt, indicating increased neovascularization (I) (CD34 stain, ×20). A: anterior; L: lateral; M: medial; P: posterior.

The following morning, the patient experienced another seizure. CT indicated a newly developed ICH in the left precentral gyrus (Figure 2A). Repeat angiography demonstrated progression of venous congestion (Figure 2B). Given the rapid clinical deterioration, transarterial embolization of the left MMA with n-butyl cyanoacrylate (NBCA) was performed the same day (Figures 2C, 2D). Because of a small residual shunt flow from the contralateral MMA, a craniotomy for surgical disconnection and resection of the fistulous point was undertaken the next day (Figures 3A, 3B). Intraoperatively, an occluded bridging vein draining the SSS was identified posterior to the cortical vein contiguous with the shunt point (Figure 3C). Postoperatively, cognitive dysfunction, too severe to assess preoperatively, markedly improved, with Hasegawa Dementia Scale-Revised (HDS-R) scores of 13/30 on day one, 19/30 on day three, and 28/30 on day eight [9].

Postoperative angiography confirmed complete obliteration of the arteriovenous shunt. The patient was discharged one month later with a modified Rankin Scale score of 1 [10]. Histopathological evaluation revealed no inflammatory changes and no remnants of venous lakes or dural veins contiguous with the SSS. These findings suggest that the shunt had formed within a normal dural arteriovenous network (Figures 3D-3I). 

## Discussion

We described a case of an anterior convexity dAVF, classified as Borden type Ⅲ and Cognard type Ⅲ, that presented with progressive aphasia and higher cortical dysfunction. Within 24 hours of admission, the patient developed ICH with seizures and significant worsening of venous congestion. This rapid clinical deterioration was likely attributable to two factors: (1) the anterior location of the lesion at the coronal suture, and (2) a cortical venous reflux pattern characterized predominantly by pial venous reflux (PVR) rather than bridging venous reflux (BVR).

Convexity dAVFs can present with seizures or intracranial hemorrhage, including both ICH and SDH [5-7,11,12]. The present case involved an anterior lesion at the coronal suture level manifesting as ICH. Few studies have directly investigated the relationship between lesion location and hemorrhagic pattern in relation to venous anatomy. Brockmann et al. analyzed 30 CT venographies and found that most bridging veins (74%) drained into the SSS at or distal to the coronal suture [13]. Similarly, Han et al. reported in both anatomical dissections and digital subtraction angiography images that tributary veins draining into the SSS were sparse around the coronal suture and near the confluence [14]. Furthermore, anterior hypoplasia or aplasia of the SSS has been reported [15], limiting drainage pathways in the anterior region. Taken together, these findings suggest that the anterior convexity region has inherently fewer direct drainage routes into the SSS, thereby restricting shunt outflow and reducing compensatory capacity for shunt outflow. Consequently, venous pressure may rise steeply, predisposing anterior convexity dAVFs to ICH. In the present case, intraoperative findings confirmed occlusion of a bridging vein dorsal to the shunt point that connected to the SSS, suggesting that the loss of this compensatory drainage route contributed to the rapid deterioration of venous outflow and ICH development. By contrast, parietal lesions often have multiple bridging veins, allowing the shunt flow to be diverted into the SSS and thereby buffering venous hypertension. However, this hemodynamic stress may predispose to SDH. Table 1 also includes an occipital-confluence lesion presenting with ICH [16], which may similarly be explained by these anatomical considerations.

**Table 1:** Summary of convexity dural arteriovenous fistula cases: lesion location, presentation, and venous drainage. ICH: intracerebral hemorrhage; N/A: not available; NBCA: n-butyl cyanoacrylate; PVR: pial venous reflux; SDH: subdural hematoma; TAE: transarterial embolization; Lt: left; Rt: right. PVR (Yes): pial venous reflux present; (No): absent.

Author, year (ref.)	Age	Sex	Symptom	Side	Location	Hemorrhagic manifestations	Venous congestion	PVR	Treatment	Clinical outcome
Hori et al., 2007 [11]	41	M	Consciousness disturbance, hemiparesis	Lt.	Parietal	ICH	Yes	Yes	Disconnection	N/A
Kohyama et al., 2009 [12]	60	M	Headache	Lt.	Parietal	SDH	No	No	TAE with NBCA	Good
Ogawa et al., 2010 [5]	27	M	Headache	Lt.	Parietal	SDH	N/A	No	Resection	Good
Saito et al., 2014 [16]	56	M	Headache	Rt.	Occipital	ICH + SDH	Yes	Yes	TAE with NBCA and resection	Good
Nagm et al., 2016 [17]	53	M	Chronic headache	Lt.	Parietal	(-)	N/A	No	Resection	N/A
Yamauchi et al., 2019 [7]	29	M	Headache	Lt.	Parietal	SDH	N/A	No	TAE with NBCA and resection	Good
Korai et al., 2022 [2]	63	M	None	Lt.	Parietal	(-)	N/A	No	TAE with Onyx	N/A
	42	F	Headache	Lt.	Parietal	(-)	N/A	Yes	TAE with NBCA	N/A
	36	M	Convulsion	Lt.	Temporal	(-)	N/A	N/A	TAE with NBCA	N/A
	73	M	Convulsion	Lt.	Occipital	(-)	N/A	N/A	TAE with Onyx	N/A
Tatezuki et al., 2024 [6]	19	M	Headache	Lt.	Parietal	SDH	No	No	TAE with coil and NBCA	Good
Present case	80	M	Convulsion	Lt.	Frontal	ICH	Yes	Yes	TAE with NBCA and resection	Good

The frequency of aggressive convexity dAVF is high, ranging from 43% to 52% [3,18]. Even within Borden type Ⅲ lesions, the risk of severe presentation may vary depending on lesion location and patterns of retrograde venous drainage. Hori and Nagm et al. suggested that physiological dAVF shunts may spontaneously expand and directly connect to cortical veins, forming convexity dAVFs without requiring occlusion of adjacent veins [8,17]. Our case supports this hypothesis, as histopathology revealed no remnants of dural veins connecting the shunt to the SSS. This finding suggests that convexity dAVFs may often be accompanied by CVR from their earliest formation stage, with venous drainage patterns influencing clinical course and the risk of severe progression. Previous reports focusing on CVR patterns have further refined risk stratification. Huang et al. categorized retrograde drainage as either BVR or PVR and demonstrated that PVR was associated with ICH and intracranial venous edema [19]. Zhao et al. also reported that PVR may increase the acute clinical deterioration risk [20]. These findings highlight the importance of qualitative CVR assessment in risk evaluation. Although limited research exists on how venous reflux patterns vary by fistula location, anterior convexity dAVFs may be more likely to exhibit predominant PVR because of the naturally sparse bridging vein distribution. Consequently, even within the same Borden type Ⅲ category, anterior lesions may present more aggressively than parietal lesions. Borden type Ⅲ dAVFs with hemorrhage carry a high risk of rebleeding within the first two weeks, for which early treatment is strongly recommended [21,22]. In our case, the patient's clinical status deteriorated rapidly over an extremely short period, and favorable functional outcomes were achieved through prompt MMA embolization followed by shunt disconnection. Nonetheless, prolonged venous congestion has been reported to cause irreversible structural brain damage due to white matter edema and demyelination [23]. Therefore, in anterior convexity dAVFs with a predominant PVR pattern, urgent treatment is crucial once symptoms develop. Moreover, even incidentally detected cases warrant close monitoring for subtle radiological changes, with early intervention considered at the first sign of symptom onset.

## Conclusions

Convexity dAVFs may inherently exhibit CVR from their earliest formation stage. When located near the coronal suture, limited bridging vein drainage to the SSS may predispose to a predominant PVR pattern. These anatomical features suggest that anterior convexity dAVFs carry a relatively higher risk of clinical deterioration than parietal lesions. Consequently, anterior convexity dAVFs with a predominant PVR pattern may follow a distinct clinical course and warrant prompt treatment upon symptom onset or even proactive intervention in asymptomatic cases with radiological evidence of progression. However, because this report describes a single case, these interpretations should be viewed with caution, and further case accumulation will be necessary.
